# Type 2 Diabetes Mellitus and Alzheimer’s Disease: Shared Molecular Mechanisms and Potential Common Therapeutic Targets

**DOI:** 10.3390/ijms232315287

**Published:** 2022-12-04

**Authors:** Rim Hamzé, Etienne Delangre, Stefania Tolu, Manon Moreau, Nathalie Janel, Danielle Bailbé, Jamileh Movassat

**Affiliations:** 1 Team Biology and Pathology of the Endocrine Pancreas, Unité de Biologie Fonctionnelle et Adaptative, CNRS, Université Paris Cité, F-75013 Paris, France; 2 Team Degenerative Process, Stress and Aging, Unité de Biologie Fonctionnelle et Adaptative, CNRS, Université Paris Cité, F-75013 Paris, France

**Keywords:** diabetes, Alzheimer’s disease, insulin resistance, tau, Aβ peptide, insulin deficiency, insulin secretion, glycogen synthase kinase 3, DYRK1A

## Abstract

The global prevalence of diabetes mellitus and Alzheimer’s disease is increasing alarmingly with the aging of the population. Numerous epidemiological data suggest that there is a strong association between type 2 diabetes and an increased risk of dementia. These diseases are both degenerative and progressive and share common risk factors. The amyloid cascade plays a key role in the pathophysiology of Alzheimer’s disease. The accumulation of amyloid beta peptides gradually leads to the hyperphosphorylation of tau proteins, which then form neurofibrillary tangles, resulting in neurodegeneration and cerebral atrophy. In Alzheimer’s disease, apart from these processes, the alteration of glucose metabolism and insulin signaling in the brain seems to induce early neuronal loss and the impairment of synaptic plasticity, years before the clinical manifestation of the disease. The large amount of evidence on the existence of insulin resistance in the brain during Alzheimer’s disease has led to the description of this disease as “type 3 diabetes”. Available animal models have been valuable in the understanding of the relationships between type 2 diabetes and Alzheimer’s disease, but to date, the mechanistical links are poorly understood. In this non-exhaustive review, we describe the main molecular mechanisms that may link these two diseases, with an emphasis on impaired insulin and IGF-1 signaling. We also focus on GSK3β and DYRK1A, markers of Alzheimer’s disease, which are also closely associated with pancreatic β-cell dysfunction and type 2 diabetes, and thus may represent common therapeutic targets for both diseases.

## 1. Introduction

Diabetes mellitus is a metabolic disease characterized by chronic hyperglycemia that arises from the impaired secretion and/or action of insulin in its target tissues. Diabetes is the most common metabolic disorder [1], with 537 million people affected worldwide in 2021 according to the International Diabetes Federation (IDF), compared to 108 million cases in 1980. This represents more than 1 in 10 adults living with diabetes. The IDF and the World Health Organization (WHO) expect this figure to increase to 643 million diabetics by 2030, due to increasing exposure to risk factors. Thus, diabetes represents a major public health issue [2,3].

Clinically, there are two main forms of diabetes. Type 1 diabetes, accounting for 10% of cases [1], arises from the autoimmune destruction of pancreatic β cells, leading to a progressive loss of insulin secretion [4]. Type 2 diabetes (T2D), which constitutes 90% of diabetes cases [4], is a multifactorial disease resulting from the interaction between genetic predispositions and environmental risk factors such as a sedentary lifestyle; nutritional imbalance; stress; and environmental pollutants such as bisphenols, dioxin and pesticides [5,6], and air pollution [7]. T2D results from peripheral insulin resistance in its target tissues, including the liver, muscle, and adipose tissue. β cells attempt to compensate for insulin resistance by increasing their number and their individual secretion. However, at some point the compensation capacity is exceeded, and β-cell exhaustion and eventually β-cell apoptosis occur, leading to partial insulin deficiency and subsequent hyperglycemia [8,9,10].

Several serious long-term complications are associated with diabetes. Most of them are the consequence of microvascular (retinopathy and nephropathy) or macrovascular (lower limb arteriopathy, cardiovascular accidents, and stroke) diseases. Several other co-morbid conditions are also associated with diabetes, especially comorbidities related to the central nervous system (CNS), including cognitive deficits, depression, and neurodegenerative diseases, especially Alzheimer’s disease (AD) [1,4,11]. In the 1990 s, the Rotterdam study provided the first epidemiological evidence of the doubling of the risk of dementia and AD in people with T2D, with a relative risk of 1.9 (95% confidence interval = 1.3–2.8) [12]. Since then, many clinical and epidemiological studies have confirmed these data, showing a strong association between T2D and AD [13,14,15,16,17]. Conversely, glucose intolerance and T2D are also common in AD patients and animal models [18,19,20]. Thus, the relationship between T2D and AD is likely bidirectional.

AD, the leading cause of dementia in the elderly [21], is a progressive neurodegenerative disease related to neuronal loss affecting the hippocampus and neocortical areas [22]. In 2020, more than 55 million people were affected by dementia worldwide. With the aging of the population, the number of patients is expected to almost double every 20 years to reach 78 million by 2030 and 139 million by 2050, as estimated by WHO [23,24]. There are two forms of AD: the familial form characterized by an early onset of symptoms, around the age of 50, due to an autosomal dominant inheritance of mutations in the genes encoding the amyloid precursor protein (APP) and presenilin 1 and 2 (PSEN1 and PSEN2) that affect the production of the amyloid beta peptide (Aβ); and the sporadic form that accounts for more than 95% of patients, and which usually develops after the age of 65 [21,25,26]. Clinically, AD is characterized by a gradual loss of memory followed by a deeper deterioration in cognitive function [21,25], manifesting as amnesia, aphasia, agnosia, and apraxia [27] and associated with behavioral and personality changes [26,28]. This ultimately results in the loss of a patient’s autonomy. Anatomically, AD is associated with two types of major lesions: (1) amyloid or senile plaques formed by the extracellular aggregation of insoluble Aβ peptide fibrils and (2) the phosphorylation and destabilization of tau (tubulin-associated unit), a microtubule-associated protein whose aggregation forms neurofibrillary tangles (NFTs) within neurons [21,25,29,30]. According to the amyloid cascade hypothesis, toxic levels of the Aβ peptide contribute to NFT formation [25,31,32]. Both these lesions lead to synaptic and neuronal dysfunction, and finally to neurodegeneration [25,26,33], causing brain atrophy in certain areas, particularly the cortex and hippocampus [21].

T2D and AD share similar etiological factors. Both conditions are degenerative, with neuronal and β-cell loss in AD and in T2D, respectively [34], and multifactorial. The pathogenesis of the sporadic form of AD involves genetic and environmental risk factors [21,28]. The most important factors are aging [25,28] and the ApoE4 (apolipoprotein E4) allele [21,26,35,36], which plays a role in the clearance of the Aβ peptide [22,37]. However, the female sex [28], and cardiovascular risk factors such as stroke, poorly controlled high blood pressure, hypercholesterolemia, obesity, and diabetes are suggested to be among the potential predisposing factors [25,28,35]. Indeed, AD could be considered a central metabolic disease due to glucose hypometabolism [38,39], associated with impaired insulin and insulin-like growth factor (IGF) signaling pathways in the brain, which are physiologically involved in energy production and neuronal survival and plasticity, and therefore play a key role in cognition and memory [40,41]. These metabolic disruptions occur years before the onset of AD [42] and worsen with AD progression [40]. The evidence for a relationship between insulin resistance and cognitive decline has led researchers to refer to AD as “type 3 diabetes” [41,43,44,45].

Given the alarming rise in the prevalence of T2D and the aging of the world’s population, the socio-economic burden of these two diseases is expected to increase in the upcoming years [17,46,47]. Therefore, understanding the molecular mechanisms that link T2D to AD in order to establish common preventive and ultimately curative measures to delay the onset and restrain the progression of these two pathologies is a major research challenge.

The aim of this review is to illustrate the common cellular and molecular pathways involved in AD and TD2, with a focus on the physiological importance of insulin/IGF-1 signaling in the brain, and its alteration during AD. We also highlight the role of the GSK3β (glycogen synthase kinase 3β)- and DYRK1A (dual-specificity tyrosine phosphorylation-regulated kinase 1A)-dependent pathological mechanisms that contribute to the development of both T2D and AD. Mechanisms such as inflammation, mitochondrial dysfunction, oxidative stress, the ApoE4 allele, and advanced glycation end-products, which are important mediators acting synergistically to induce these pathological conditions [1,48,49,50,51], are not discussed in this review.

## 2. Insulin and IGF-1: Physiological Role in the Brain

The brain uses about 25% of the total body glucose [52] to produce the energy needed to maintain normal metabolic activity, vitality, neurotransmission [53], synaptic plasticity [4], and neuronal ionic gradients [26,52]. Although initially considered an organ with insulin-independent glucose metabolism [54,55] and a high expression of insulin-independent glucose transporters GLUT1 and GLUT3 [55,56], more recently, biochemical evidence has shown that the brain is also a target organ for insulin, and the expression of insulin, insulin receptors, insulin-dependent GLUT4 [1,57] and GLUT8 transporters [55,58] has been documented in specific brain regions.

### 2.1. The Origin of Insulin in the Brain

The origin of insulin in the brain remains controversial. The insulin levels in cerebrospinal fluid (CSF) represent 25% of the plasma levels [59]. There is a correlation between plasma and CSF insulin [60], since insulin levels in the CSF increase proportionally after meals or after a peripheral insulin infusion [59], suggesting that the majority of insulin present in the brain comes from circulating insulin synthesized by pancreatic β cells [60,61]. To reach the brain, insulin crosses the blood-brain barrier (BBB) via a saturable and selective transporter in the vascular endothelium [51,60,61,62]. This hypothesis is supported by the fact that the CSF-to-serum ratio of insulin is decreased with insulin resistance [63] as well as with age [64] and during AD [65], due to a decrease in the expression of insulin carriers at the BBB, which alters its transport to the brain [60].

In addition to the pancreatic origin of insulin found in the brain, there is debate regarding the possibility of the local synthesis of insulin in the CNS. The expression of insulin mRNA has been observed in various regions of the brain in animal models [59,61]. Clinical studies in humans have also shown the production of C peptide (a short peptide part of the proinsulin molecule, which is secreted in an equimolar fashion with insulin by pancreatic β cells) in some areas [66], but this could also originate from the pancreas. Insulin mRNA has been identified in the postmortem human brain with decreased expression in those suffering from AD [43,60]. However, the cerebral origin of insulin remains controversial, and whether the amount of insulin produced in the brain is physiologically relevant is still a matter of debate [59].

### 2.2. Expression of Insulin and IGF Receptors in the Brain

Insulin receptors are expressed in neurons and glial cells [28,67], with different levels of expression in distinct regions of the brain [60]. Their density is particularly high in the hypothalamus, hippocampus, cerebral cortex, cerebellum, and olfactory bulb [15,28,55,60,67], which are all critical areas for metabolic control and cognition [10]. Like the insulin receptor, the IGF-1 and IGF-2 receptors are expressed in the brain, especially in the hippocampus, cerebral cortex, and thalamus [55,59]. Some effects of insulin may be mediated through its binding to the IGF-1 receptor [68], and, conversely, IGF-1 and IGF-2 may also bind to the insulin receptor but with a lower affinity [26].

### 2.3. Insulin and IGF Signaling and Actions in the Brain

**Figure 1:**
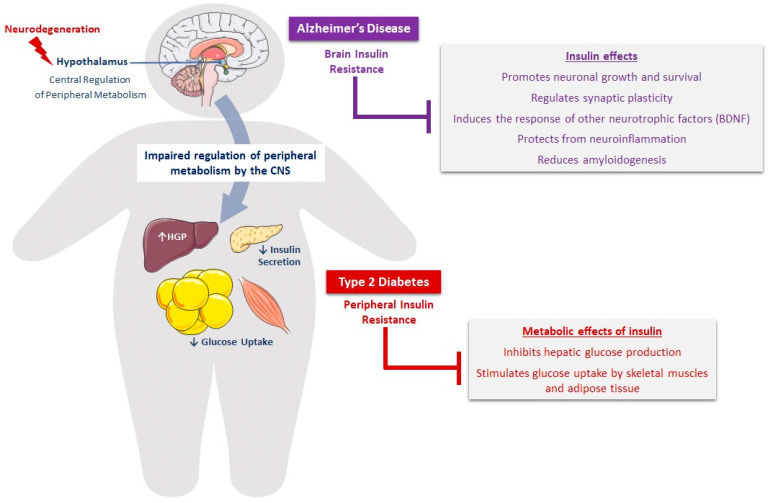
Defective insulin signaling as a major molecular mechanism linking T2D and AD. The pathophysiology of T2D implicates peripheral insulin resistance, leading to decreased glucose uptake by skeletal muscles and adipose tissue and increased hepatic glucose production (HGP). Because insulin is a key neurotrophic and neuroprotective factor, brain insulin resistance would contribute to the pathogenesis of AD. Conversely, the neurodegeneration that occurs in the hypothalamus leads to the impaired regulation of peripheral metabolism and defective insulin secretion by pancreatic β cells.

Beyond the canonical role of insulin in the regulation of peripheral energy metabolism primarily in the hypothalamus [61], insulin is a key neurotrophic and neuroprotective factor in the brain [15,30], promoting neuronal growth and survival, thus making it an important modulator of cognition and memory [61,69,70] (Figure 1). IGF-1 is also an important trophic factor for neurogenesis, myelination [71], synaptogenesis [72], and especially for neuronal protection and regeneration following injury [30,70].

The effect of insulin on cognition could also be mediated by the regulation of brain glucose metabolism in regions important for learning and memory, such as the hippocampus and cortex [1,70,73,74]. This action is mediated by increasing the expression and translocation of GLUT4 and thereby the uptake of glucose [74,75]. In addition, insulin plays a key role in synaptic plasticity by inducing long-term potentiation through the regulation of the expression of the N-methyl-D-aspartate (NMDA) glutamate receptor, and by stimulating its membrane recruitment in excitatory synapses in hippocampal neurons [59,69,76]. This increases the neuronal calcium influx, allowing for a prolonged reinforcement of synaptic communication between neurons [70,77]. Insulin also promotes the internalization of the AMPA (α-amino-3-hydroxy-5-methyl-4-isoxazolepropionic acid) glutamate receptor via the PI3K/PKC pathway, which induces long-term depression essential for both memory consolidation and flexibility [28,59,78,79]. Furthermore, insulin is implicated in the recruitment of the GABA (gamma-aminobutyric acid) receptor to the postsynaptic membrane, thus regulating inhibitory synapses [28,59,70,80,81]. It is well-established that GABAergic signaling is decreased in AD [82]. A recent study has shown that synaptic GABA-activated currents were reduced in hippocampal slices of an AD mouse model, while the extrasynaptic currents were, on the contrary, increased compared to wild-type mice. Interestingly, the application of near-physiological concentrations of insulin (1 nmol/L) to hippocampal slices of aged tg-APPSwe normalized both GABAergic currents to control levels, enhancing the synaptic transmission and decreasing the extrasynaptic transmission. These results indicate that the insulin remodeling of GABA signaling is essential to maintain normal neural circuits and could restore the excitatory–inhibitory imbalances in AD [83]. Insulin has been shown to modulate the levels of neurotransmitters such as acetylcholine and norepinephrine that impact cognition [26,74], and it also regulates the expression of genes necessary for long-term memory consolidation [1,69]. Additionally, insulin regulates the number of synapses [84] and stimulates the formation of dendrites and the expression of PSD95 (postsynaptic density protein 95), a protein necessary for the formation of the postsynaptic junction [59,85]. Glial cells are also dependent on insulin, as it stimulates glial cell proliferation as well as oligodendrocyte survival, differentiation, and myelination [59,60,86]. Importantly, insulin signaling induces cell responses to other neurotrophic factors. Indeed, the intracerebroventricular (i.c.v) injection of insulin in adult rats increased BDNF/TrkB (brain-derived neurotrophic factor/tropomyosin receptor kinase B) signaling in the hippocampus and improved their spatial memory performance during the Morris water maze test [87]. BDNF in turn plays a crucial role in synaptic maturation, connection, and plasticity and neuronal regeneration [68,88,89,90]. Neuronal survival is also promoted by insulin [60]. This occurs through the activation of Akt, which inhibits apoptosis through the post-translational modulation of several proteins involved in cell survival [28]. Finally, insulin protects neurons from neuroinflammation. In line with these effects, it has been shown that i.c.v insulin administration modulates the secretion of inflammatory cytokines by astrocytes induced by intraperitoneal lipopolysaccharide injection and reduces oxidative stress by preserving mitochondrial function [91].

Thus, changes in insulin levels and/or signaling in the brain could lead to neuronal loss and synaptic dysfunction associated with cognitive decline and the disruption of peripheral metabolism [10] (Figure 1). These important effects of insulin in the brain are supported by a number of studies in animal models of AD [15,92,93]. Further, intravenous or intranasal insulin administration has led to memory improvement in humans and animals [26,94,95,96], suggesting that impaired insulin signaling could be one of the main defects linking AD to T2D.

## 3. Molecular Mechanisms Linking T2D to AD

T2D and AD share many pathophysiological features, and some factors seem to mediate the dialogue between these two conditions.

### 3.1. Cerebrovascular Abnormalities in Diabetes and AD

Numerous studies support the hypothesis that an imbalance between Aβ production and clearance initiates AD by promoting Aβ accumulation in the CNS [33,97]. Nevertheless, while the early-onset form of AD arises from the genetic overproduction of Aβ [98,99], the sporadic form is rather the result of impaired Aβ clearance [100]. A study has shown that in late-onset AD, the Aβ clearance rate is 30% slower (5.3%/hour for AD patients versus 7.6%/hour for control subjects, *p* = 0.03) [101]. Physiologically, Aβ efflux from the brain is mediated by several pathways [102,103]. Aβ is transported across the endothelial cells of the BBB through LRP1 (low-density lipoprotein receptor-related protein 1) [104] and ABCB1 (ATP-binding cassette subfamily B member 1) transporters into the peripheral bloodstream [105]. Another important clearance pathway is the lymphatic-related pathway: as there is no conventional lymphatic system in the brain, the interstitial fluid (ISF) of the brain is drained to cervical lymph nodes along venules (glymphatic system) [106] or via perivascular circulation alongside basement membranes in capillary and artery walls [107,108]. The vascular-mediated clearance system is an active process driven by vasomotion. This force is generated by vascular smooth muscle cell contraction and relaxation cycles [109]. However, this system becomes defective with aging due to the loss of elasticity and the stiffening of the artery walls [103]. Cerebrovascular diseases evidenced by morphological abnormalities in the cerebral capillaries [110], ischemic infarcts [111], and reduced cerebral blood flow [112] have been documented in AD. These neurovascular alterations lead to hypoperfusion and chronic hypoxia and finally to neurodegeneration [113,114], and may contribute to the pathogenesis of AD, notably by impairing Aβ elimination via the vascular pathway.

Interestingly, a growing body of literature suggests that cerebrovascular disease contributes to cognitive impairment in diabetic patients [115]. In T2D, chronic hyperglycemia and oxidative stress damage the vascular endothelium and promote atherosclerosis, leading to various vascular complications [116]. T2D is associated with an increased risk of ischemic stroke and acute cerebral infarcts [117,118]. Moreover, chronic hyperglycemia leads also to the remodeling of cerebral microvascularization, evidenced by the thickening of the cerebral capillary basement membrane in diabetic animal models [119] and diabetic patients [120,121]. This thickening may alter the integrity of vascular smooth muscle cells [122] and leads to increased microvascular resistance [123]. The dysregulation of cerebrovascular function in diabetes can severely impact cerebral perfusion and function and the removal of metabolites out of the brain. More specifically, by altering brain vessel integrity and elasticity, T2D may impair the vascular-mediated Aβ clearance system and thus contribute to Aβ deposition in the brain. Therefore, cerebrovascular disease could be a common mechanism linking T2D and AD.

### 3.2. Alteration of Insulin and IGF-1 Signaling in the Brain

#### 3.2.1. Insulin/IGF-1 Resistance, Neurodegeneration, and Cognition

Even when vascular risk factors are controlled, the risk of developing AD in diabetic patients remains high, suggesting that there are non-vascular mechanisms involved in the pathogenesis of AD [30,124]. Compared to normoglycemic patients, the progression from mild cognitive impairment to AD is greater in patients with impaired blood glucose levels [125,126]. Given the importance of insulin in cognition, the deregulation of its signaling in the brain may be responsible for cognitive defects in patients with T2D and AD [54]. A number of postmortem studies have indicated that resistance to insulin and IGF-1, with the aberrant activation of their signaling pathway components, as well as reduced insulin/IGF-1 levels as neurotrophic factors, can be detected in the brains of AD patients [43,127,128,129,130], and these abnormalities are more severe in areas involved in cognitive performance, particularly in the hippocampus [131]. In fact, the early stages of AD, potentially decades before the development of symptoms, are characterized by deficits in cerebral carbohydrate metabolism that worsen with disease progression [40,132,133]. Likewise, insulin-resistant elderly people [26,134], T2D patients with mild cognitive impairment [135,136], and even prediabetic patients with normal cognitive function [49,134] show brain hypometabolism quantified by a decreased uptake of [^18^F]-FDG (18-fluorodeoxyglucose) detected by PET (positron emission tomography) imaging. Interestingly, the central impairment of glucose metabolism has been associated with insulin and IGF-1 resistance [40,132,133,137] and is mostly apparent in the frontal, parietotemporal, and cingulate cortices [134], indicating that insulin resistance affects the same regions as those affected by AD [54], and suggesting a link between central insulin resistance and this neurodegenerative disease.

Notably, AD patients have decreased insulin receptor expression and activation in the brain [43,59] and reduced CNS levels of total IRS (insulin receptor substrate) mRNAs [43,60] and PI3K and phospho-Akt levels [138,139]. These patients have lower insulin levels in the CSF than healthy control subjects, while their fasting plasma insulin levels are high [59]. The CSF/plasma ratio of insulin is therefore reduced [34,77]. Peripheral insulin resistance is more common in patients with AD than in healthy aging subjects [10,18]. It is accompanied by chronic compensatory hyperinsulinemia in an attempt to maintain glucose homeostasis [26]. However, hyperinsulinemia, with or without T2D, negatively affects the availability and action of insulin at the central level by causing the compensatory downregulation of insulin carriers at the BBB. Consequently, the amount of insulin passing into the brain decreases [26,140]. An increase in IRS-1 inhibitory serine phosphorylation instead of tyrosine phosphorylation is a marker of insulin resistance. This phenomenon is also observed in AD patients [54,131,141] and in the hippocampus of transgenic mouse models of AD, such as the APP/PS1 model [92,142]. These abnormalities are accompanied by the suppression of the activation of downstream kinases and the expression of genes regulated by insulin and IGF signaling pathways, in particular a reduction in the choline acetyltransferase responsible for the synthesis of acetylcholine, a neurotransmitter important for cognition, and a reduction in glyceraldehyde-3-phosphate dehydrogenase (GAPDH), which is involved in metabolic functions [143]. Insulin and IGF-1 resistance in the brain activates pro-apoptotic, pro-inflammatory, and pro-APP-Aβ cascades and affects the expression and metabolism of the tau protein by promoting oxidative stress, the generation of reactive oxygen species (ROS), mitochondrial dysfunction, and DNA damage. All these events contribute to neurodegeneration [40,144].

In addition, the induction of diabetes in mouse models of AD leads to the exacerbation of memory and learning impairments and an increase in amyloid deposition [10,145]. Experimental animal models of T2D, such as HFD (high-fat diet) rodents, obese *ob/ob* mice, obese and diabetic *db/db* mice, or Zucker rats with leptin resistance, also exhibit AD-like alterations such as an increase in tau phosphorylation, a deficit of neuroplasticity evidenced by an impairment in long-term potentiation, and decreased neurogenesis [126,129,146]. The expression of the neurotrophic factor BDNF is also decreased in the hippocampus of obese rodents [146,147,148]. These conditions lead to decreased synaptic plasticity, probably due to insulin resistance [146]. In line with this, intranasal insulin administration in AD patients slows down cognitive decline [59,96], and insulin-sensitizing antidiabetic medications such as metformin and PPAR-γ agonists (peroxisome proliferator-activated receptors) [10], as well as other treatments such as GLP-1 (glucagon-like peptide-1) analogues and DPP4 (dipeptidyl peptidase 4) inhibitors [126,146], prevent neurodegeneration in T2D models.

#### 3.2.2. Bidirectional Relationship between Insulin/IGF-1 Resistance and Amyloidogenesis in T2D and AD

(1) Impact of insulin resistance on amyloidogenesis

AD and T2D are amyloidogenic pathologies characterized by an abnormal aggregation of the Aβ peptide and the pancreatic islet amyloid polypeptide (IAPP), also known as amylin, in the brain and in the pancreas, respectively, both contributing to cell death and the pathogenesis of these diseases [26,34,149,150,151]. Extracellular senile plaques caused by the aggregation of insoluble amyloid fibrils are an important pathological feature of AD and are involved in neurodegeneration [1,48,152]. Aβ peptide is a 4-kDa peptide that results from the proteolytic cleavage of the transmembrane protein precursor APP [48]. There are two main pathways for APP cleavage: a non-amyloidogenic pathway in which APP is cleaved by the α-secretase enzyme to produce the soluble APPα fragment (sAPPα), which is neuroprotective and does not generate Aβ; and an amyloidogenic pathway where APP is cleaved at multiple sites, successively by a β-secretase, the BACE-1 enzyme (β-site APP-cleaving enzyme 1), and a γ-secretase complex formed by the presenilins, resulting in the formation of different lengths of the Aβ peptide such as Aβ40 and Aβ42 [17,34,153]. The Aβ peptide plays a physiological role in synaptic plasticity and neuronal survival, but an imbalance between its production and clearance promotes its accumulation and subsequent toxicity [1]. Being more hydrophobic, the Aβ42 peptide is more apt to aggregate than the Aβ40 form [25,153].

Numerous in vitro studies indicate an association between altered insulin signaling and the amyloid cascade [54,154]. This may explain why diabetic patients are more likely to develop AD. Insulin modulates APP precursor expression and metabolism in the brain in order to maintain the balance between Aβ production and degradation [28]. In normal conditions, insulin and IGF-1 inhibit Aβ peptide production by inactivating the GSK3β enzyme via its phosphorylation by Akt and by inhibiting the translation of mRNAs of the BACE-1 enzyme and its substrate APP. Activated Akt also inhibits GSK3α, an isoform which stimulates the production of Aβ by γ-secretase [149,155,156,157]. Finally, insulin increases the expression of α-secretase, which mediates the cleavage of APP by the non-amyloidogenic pathway [158]. In addition, insulin and IGF-1 prevent the intracellular accumulation of Aβ peptide by accelerating its trafficking from the Golgi/trans-Golgi network to the plasma membrane to allow its extracellular secretion [26,141,157,159]. They also prevent the accumulation of the Aβ peptides by stimulating the transport of Aβ-binding carrier proteins in the brain, in particular albumin and transthyretin [157,160,161]. Insulin interferes with the extracellular proteolytic degradation of the Aβ peptide by the insulin-degrading enzyme (IDE), a metalloprotease also responsible for the catabolism of insulin and IGF-1 [28,48,162,163] and whose expression increases following Akt activation by the insulin receptor, thus acting as a negative-feedback mechanism [30,164]. mRNA levels, protein levels, and the activity of IDE are decreased in AD brains [165,166,167]. In an insulin-resistance state, the decreased activation of the PI3K/Akt pathway reduces IDE activation [30], and hyperinsulinemia competitively inhibits IDE. As a result, Aβ degradation decreases, promoting its neurotoxic accumulation and the development of AD [48,49,126,157,168].

Not only does Aβ peptide clearance decrease, but Aβ peptide production (1–40 and 1–42) increases when these signaling pathways are impaired [40,169], thereby promoting the aggregation of monomers into large oligomeric fibrils, or their organization into cross-β-sheet units that form amyloid fibrils in senile plaques [152]. Given the importance of insulin in the regulation of amyloidogenesis, the brains of patients with T2D are more susceptible to the toxicity of Aβ. In keeping with these observations, insulin administration attenuates amyloid accumulation, protects synapses from Aβ toxicity, and improves cognitive performance in animal models of AD and in humans, illustrating the role of insulin signaling in amyloidogenesis [15,170,171].

(2) Impact of amyloidogenesis on insulin/IGF-1 signaling

On the other hand, APP-Aβ oligomers are toxic and can induce or exacerbate neuronal insulin resistance by the abnormal activation of the TNF-α/JNK (tumor necrosis factor alpha/c-Jun N-terminal kinase) pathway, leading to the serine phosphorylation of IRS-1 [92,154,172,173], and by the induction of mitochondrial oxidative stress [174,175,176]. The TNF-α/JNK pathway is also activated in T2D, leading to peripheral insulin resistance and contributing to pancreatic β-cell apoptosis and increased oxidative stress [45,157,177,178]. APP-Aβ oligomers also disrupt insulin signaling by competitively binding to its receptor. This reduces insulin’s affinity for its receptor, and results in desensitization [10,149,167,179]. The decreased autophosphorylation of the insulin receptor and the subsequent alteration of its downstream cascade could lead to synaptotoxicity by altering long-term potentiation and neuronal dysfunction, and consequently to memory impairment [26,40,149,173,180,181]. Indeed, the exposure of primary hippocampal neurons to Aβ oligomers in vitro causes the loss of their sensitivity to insulin, the inhibitory phosphorylation of IRS-1, and the significant removal of insulin receptors from the plasma membrane of dendrites [15,170,182]. APP/PS1 mice overexpressing Aβ also exhibit impaired insulin signaling in the hippocampus, evidenced by an increase in IRS-1 phospho-serine levels [92]. Furthermore, the hippocampal injection of Aβ oligomers in rats alters insulin signaling by decreasing Akt phosphorylation and plasma membrane GLUT4 translocation [126,183]. Conversely, IGF-1 blocks amyloid toxicity by activating survival signaling pathways via ERK and PI3K/Akt and downstream phospho-BAD and transthyretin involved in Aβ clearance, explaining the limited neurotoxicity of Aβ peptides in APP-overexpressing transgenic mice in the presence of IGF-1 [30,184,185,186]. Intriguingly, the expression of the IGF-1 receptor is increased in areas surrounding amyloid plaques in the brain. This is probably a compensatory mechanism for insulin deficiency [10,187].

In addition to the brain, APP is expressed in key tissues involved in the regulation of glucose metabolism, such as the liver, skeletal muscle, adipose tissue, and the pancreas [10,188,189]. T2D patients have amyloid deposits in their pancreas similar to the senile plaques found in the brains of patients with AD. This is associated with the loss of β-cell mass and function [48,149,190,191]. These deposits are formed by the aggregation of amylin, a 37-amino acid peptide derived from the proteolytic cleavage of an 89-amino acid precursor [190,192,193]. Amylin fibrils are similar to those of the Aβ peptide [48,194]. Physiologically, IAPP is co-secreted with insulin by pancreatic β cells [188,195] and is implicated in the regulation of postprandial glycemia, gastric emptying, and food intake, and in the inhibition of glucose-stimulated glucagon secretion [13,191]. Under normal conditions, IAPP does not aggregate, but its structure is altered when exposed to a disturbed chemical environment such as a high pH and low calcium concentration, conditions associated with β-cell damage, even before the onset of T2D [34]. Interestingly, high levels of the Aβ peptide are also present in the pancreas of type 2 diabetic patients [190]. Some studies also show the deposition of IAPP in the brains of patients with AD, independently of the Aβ peptide [188,196]. Similarly, amylin deposition was found in brain vessels of T2D patients [197]. The inoculation of pancreatic IAPP aggregates into the brains of transgenic mice with AD led to the worsening of the memory deficit compared to untreated mice. At this level, IAPP oligomers damage the membrane permeability in neurons, induce the production of ROS, and alter calcium homeostasis [10]. It is important to note that just like the induction of neuronal death by Aβ oligomers, amylin aggregates induce β-cell apoptosis [149], suggesting that both peptides have similar cytotoxic mechanisms. In addition, the co-localization of amylin and Aβ peptides in the brain suggests that amylin contributes to the metabolic risk of AD [26].

Together, these observations imply that insulin resistance and Aβ toxicity have a bidirectional relationship and may constitute a vicious circle for the aggravation of dysmetabolic and neurodegenerative processes in T2D and AD, respectively.

#### 3.2.3. Insulin and IGF-1 Resistance, GSK3β, and Tauopathy in T2D and AD

Insulin and IGF-1 demonstrate neuroprotective actions by reducing the activity of the GSK3 enzyme and thereby the phosphorylation of tau proteins in cultured neurons [198], thus preventing the formation of intraneuronal NFTs [30,139,154,199]. The GSK3 enzyme is a constitutively active serine/threonine kinase that exists as two isoforms (α and β) ubiquitously expressed in tissues, and with similar biochemical properties. They have an N-terminal inhibitory phosphorylation site (Ser21 for α and Ser9 for β) and a phosphorylation-facilitating site (Y279 for α and Y216 for β) in their catalytic domain. GSK3 α and β are involved in various cellular processes such as glycogen metabolism, gene transcription, cell apoptosis, neuronal function, and microtubule stability [158,200,201,202]. Their activity can be inhibited by a variety of stimuli, including the insulin, growth factor, and Wnt pathways [158,203,204].

**Figure 2:**
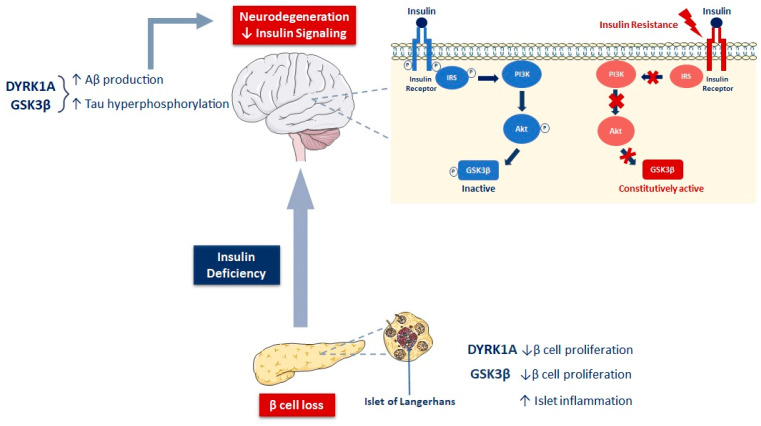
DYRK1A and GSK3β enzymes: potential molecular actors implicated in pancreatic β-cell loss and neurodegeneration. Under physiological conditions, insulin inhibits GSK3β activity by phosphorylating it via the PI3K/Akt pathway. In the case of insulin resistance, GSK3β remains dephosphorylated and constitutively active, resulting in the hyperphosphorylation of tau. The aberrant activation of DYRK1A and GSK3β in the brain increases Aβ peptide production and causes the hyperphosphorylation of tau, resulting in the formation of amyloid plaques and neurofibrillary tangles, respectively. These aggregates cause neurodegeneration and induce the alteration of brain insulin signaling. In the endocrine pancreas, both DYRK1A and GSK3β inhibit β-cell proliferation, and GSK3β is associated with the inflammation of the islets of Langerhans. This leads to β-cell loss and insulin secretion deficiency, further aggravating the impaired insulin signaling in the brain.

GSK3 phosphorylates a large number of substrates, including several neuronal proteins directly related to AD, in particular tau, involved in the regulation of microtubule stabilization and dynamics and in axonal transport [158,202,205]. Tau mRNA undergoes alternative splicing in the adult brain, which results in six isoforms containing either three (3R) or four (4R) microtubule-binding repeat domains [202,206,207]. Healthy brains contain similar levels of the 3R and 4R isoforms [202,208]; the 4R isoform binds and modulates microtubules more effectively [190,209]. Normally, the phosphorylation/dephosphorylation of tau is a dynamic process important for its functionality. Tau phosphorylation induces its release from microtubules to facilitate the axonal transport of vesicles, while its dephosphorylation induces its re-binding to tubulins [126,202,210,211]. Insulin and IGF-1 inhibit the activation of GSK3β by phosphorylation on serine 9 via the PI3K/Akt pathway, thus limiting its ability to phosphorylate tau and promoting the binding of tau to microtubules [126,139] (Figure 2). Akt activation appears to be particularly important because it also inactivates protein phosphatase 2A (PP2A) to keep GSK3 phosphorylated [158]. In the case of insulin or IGF-1 resistance, as in T2D, due to impaired Akt activation, GSK3 remains dephosphorylated and constitutively active, resulting in the hyperphosphorylation of tau [48,49,212] (Figure 2). Hyperphosphorylated tau folds abnormally and becomes more prone to self-aggregation into insoluble paired helical filaments (PHFs) [126,201,213]. PHF-tau is the main component of NFTs. GSK3β levels are increased in the brains of AD patients [214], and immunohistochemical studies have shown the co-localization of GSK3 in the PHF-tau aggregates [203,215]. Apart from hyperphosphorylation, impaired insulin and IGF signaling alters the expression of the gene encoding tau. This results in the insufficient production of normal soluble tau proteins in favor of the accumulation of insoluble fibrils of hyperphosphorylated tau [40]. These NFTs disturb the cytoskeletal network and axonal transport. Eventually, the ubiquitination of hyperphosphorylated tau [216,217,218], associated with the dysfunction of the ubiquitin–proteasome system [219], leads to the development of oxidative stress. These abnormalities ultimately lead to synaptic and mitochondrial dysfunction and progressive neurodegeneration in AD [40].

In addition, GSK3 negatively regulates Wnt signaling, which is an important pathway involved in synaptic plasticity. Thus, in cerebral insulin resistance, the hyperactivation of GSK3 participates in the impairment of synaptic plasticity [143]. Aβ peptides can also induce GSK3 dysregulation [158]. Indeed, since the insulin [180] and Wnt [220] signaling pathways are targeted by Aβ peptide toxicity, amyloid accumulation also contributes to an increased activation of GSK3β, and consequently leads to tau hyperphosphorylation, thus establishing a link between senile plaques and NFTs in AD [158,213].

Interestingly, elevated levels of hyperphosphorylated tau have been found in the islets of Langerhans of T2D patients, indicating that tau pathology is also a hallmark of T2D [190]. Tau is expressed in pancreatic β cells, where its phosphorylation/dephosphorylation plays a role in insulin trafficking and secretion [221]. Our team has significantly contributed to the establishment of the role of GSK3β as a negative regulator of β-cell growth and function. We provided the first evidence for the implication of the Wnt/β-catenin signaling pathway in the regulation of the physiological expansion of the β-cell mass during the early post-natal period [222]. Moreover, GSK3β downregulation by pharmacological or genetic modulators resulted in the stimulation of β-cell regeneration in neonatal diabetes induced by streptozotocin [222]. Likewise, in a more recent study, we reported that the local intrapancreatic knockdown of GSK3β in 90%-pancreatectomized rats promoted β-cell and exocrine cell regeneration in the remnant pancreatic tissue by stimulating cell proliferation and neogenesis [223]. Other studies have shown that transgenic mice overexpressing a constitutively active form of GSK3β exhibit impaired glucose tolerance and insulin secretion in response to glucose and decreased β-cell proliferation and mass compared with non-transgenic littermates [224]. Finally, in a recent study, our team provided the first evidence for the implication of GSK3β in diabetes-associated islet inflammation [225]. Together, all these data designate GSK3β as a negative regulator of β-cell mass and function and suggest that this enzyme could be a relevant target for the regenerative therapy of diabetes. It is interesting to note that the link between GSK3 and T2D diabetes has been initially established through its role in the insulin signaling pathway. GSK3β has been shown to phosphorylate IRS-1 on serine and thereby inhibit downstream insulin signaling [203,226]. Increased GSK3β activity was observed in peripheral insulin-sensitive tissues, including in the skeletal muscle of T2D patients, thus contributing to insulin resistance [224,227]. GSK3β is therefore involved in both insulin resistance and insulin deficiency, two main defects at the origin of T2D pathogenesis. Given the central role of this enzyme in both AD and T2D, GSK3 could be considered as a common target for the treatment of these interconnected pathologies (Figure 2).

### 3.3. Involvement of DYRK1A in AD and Diabetes

Another potentially important molecular actor in the pathogenesis of AD that may be linked to diabetes is DYRK1A. DYRK1A is a serine/threonine kinase and a member of the DYRK protein family that has five different isoforms. DYRK proteins are self-activated by the autophosphorylation of the tyrosine residue conserved in their activation loop and phosphorylate their substrates on serine and threonine residues [228,229,230,231]. The gene encoding DYRK1A is located on human chromosome 21, a critical genomic region involved in Down syndrome (DS) [232,233,234]. DYRK1A is an ubiquitous enzyme whose regulated expression from the fetal stage and in adulthood is essential for normal brain development and function, including neurogenesis, dendritogenesis, and synaptogenesis [230,231,233]. The aberrant overexpression of DYRK1A in DS due to the third copy of its gene [235] contributes to abnormal brain development with defective neurogenesis [236] and neurodegeneration [237]. In addition, recent studies have shown that DRK1A is involved in tau [238] and amyloid pathologies [239] associated with the early onset of AD in patients with DS [236,237,240]. Moreover, polymorphisms in DYRK1A may be associated with an increased risk of AD [241]. DYRK1A induces the pathological features of AD by phosphorylating substrates involved in different signaling pathways (Figure 2). In particular, DYRK1A phosphorylates tau on threonine (Thr) 212, a hyperphosphorylated residue in AD, but also on other residues. This phosphorylation primes tau for subsequent phosphorylation by GSK3β, which promotes the formation of NFTs [236,237,238,242]. There is also a co-localization of DYRK1A in these aggregates [243], similar to that reported for GSK3β [215]. Another substrate phosphorylated by DYRK1A is the alternative splicing factor (ASF), which controls the splicing of tau, thereby decreasing the formation of the 4R-tau isoform and increasing that of 3R-tau. The modification of the 3R-tau/4R-tau ratio alters the neuronal cytoskeleton and also triggers neurofibrillary degeneration [237,244,245]. DYRK1A overactivity also induces the pathological amyloidogenic pathway: high levels of Aβ are detected in the hippocampus of DYRK1A transgenic mice and in the brains of DS patients [236,239]. DYRK1A phosphorylates APP on Thr-668 [239] and PSEN1 on Thr-354 [236,246], which increases the proteolytic cleavage of APP by the BACE1 [247] and γ-secretase enzymes [248] and consequently the production of Aβ peptides [237]. Thus, through this mechanism, DYRK1A is also involved in amyloid plaque formation. Conversely, high levels of the Aβ peptide increase DYRK1A expression and consequently the hyperphosphorylation of tau [241]. DYRK1A is therefore an important link between Aβ and tau pathology.

Interestingly, DYRK1A is not only involved in neurodegenerative diseases but also in the proliferation of β cells [230,249,250,251] (Figure 2). Several studies show that the inhibition of DYRK1A stimulates β-cell proliferation, increases pancreatic islet mass, and improves glycemic control in diabetic mice transplanted with a marginal mass of human islets [230,252]. In addition, DYRK1A has been shown to be associated with hyperhomocysteinemia and reduced BDNF in AD [68,253], but also in patients with T2D [254,255]. As mentioned earlier, DYRK1A primes some of the substrates of GSK3β by phosphorylation on P+4 serine in the SXXXS motif, relative to the GSK3β phosphorylation site (P) [242,256].

The implication of DYRK1A in these processes points to both a direct and potentially indirect (via GSK3) negative role of this enzyme in β-cell homeostasis and insulin sensitivity. We present here a non-exhaustive list of studies using GSK3β (Table 1) and DYRK1A (Table 2) inhibitors as therapeutic approaches for diabetes and AD.

## 4. Conclusions

Although T2D and AD were initially considered two independent pathologies, substantial epidemiological arguments show an increased risk of developing AD in diabetic patients, and there is now sufficient experimental evidence of a common pathophysiological mechanism for these two diseases. Beyond its canonical role in maintaining energy homeostasis, insulin is a neurotrophic and neuroprotective factor important for maintaining cognitive function, prompting many researchers to consider AD as a metabolic disease linked to insulin deficiency. Anatomical and functional abnormalities are found in the same brain areas in T2D and AD. In this non-exhaustive review, we presented the main molecular mechanisms of degeneration, in which T2D contributes to the development and progression of AD. Targeting these pathways with antidiabetic therapies could be an approach to correct defective insulin signaling in the brain and limit neurodegeneration. Notably, the intranasal administration of insulin has been beneficial in improving memory in AD patients who do not have the ApoE4 allele. The beneficial effects of insulin-sensitizing drugs such as metformin, GLP-1 and its analogues, DPP-IV inhibitors, and thiazolidinediones are also being assessed in AD.

In this review, we also emphasized the role of two enzymes, GSK3β and DYRK1A, which have deleterious roles both in T2D and AD. The aberrant activation of these kinases results in the hyperphosphorylation of tau and the aberrant cleavage of APP, leading to amyloid accumulation. In turn, these products induce defective insulin signaling, neuroinflammation, and oxidative stress, thereby establishing a vicious cycle. On the other hand, the activation of DYRK1A and GSK3 β induces β-cell loss and dysfunction, leading to insulin deficiency, which is not only the root-cause of T2D pathogenesis, but also an important contributor to brain dysfunction. With the recent findings regarding the crucial roles of GSK3β and DYRK1A in diabetes pathogenesis, we believe that the development of potent and selective inhibitors for these targets could be of great importance to treat both T2D and AD. Given the alarming progression of these two pathologies in our aging societies, it is highly recommended to decipher the pathophysiological mechanisms linking T2D to AD, and to identify common molecular targets for their treatment.

## Figures and Tables

**Table 1:** Inhibition of GSK3β as a therapeutic approach for diabetes and AD.

Molecular Target	Disease	Experimental Model	Main Findings	References
GSK3β Inhibition	Diabetes	Zucker diabetic fatty *(fa/fa)* rats	Activation of liver glycogen synthesis and improvement in glucose disposal. Downregulation of key enzymes of gluconeogenesis and attenuation of basal endogenous glucose production.	[257]
		Zucker prediabetic fatty *(fa/fa)* rats	Decreased fasting plasma insulin and free fatty acid levels. Increased muscle IRS-1 dependent insulin signaling; enhancement in insulin-stimulated glucose transport above basal in skeletal muscle. Enhancement in whole-body glucose tolerance and in sulin sensitivity index.	[258]
		Neonatal streptozotocin-induced diabetes in rats	Stimulation of β-cell regeneration.	[222]
		90%-pancreatectomized Wistar rats	Induction of β-cell proliferation and neogenesis. Induction of ductal and acinar cell proliferation.	[223]
		Diabetic Goto–Kakizaki rats	Improvement in insulin sensitivity. Reduction in islet inflammation. Improvement in glucose-induced insulin secretion.	[225]
	AD	JNPL3 transgenic mice overexpressing mutant human tau	Reduction in insoluble tau aggregation in the cortex. Reduction in tau phosphorylation at specific epitopes (serine 202 and serine 396/404). Reduction in axonal degeneration.	[259]
		Patients with amnestic mild cognitive impairment	Reduction in CSF p-tau levels. Attenuation of cognitive decline.	[260]
		5XFAD mouse model of AD	Restoration of brain lysosomal acidification, enabling Aβ load clearance. Reduction in cerebral amyloid deposits.	[261]
		P301L human tau transgenic mice	Reduction in tau hyperphosphorylation in thecortex and spinal cord.	[262]
		Rat embryonic hippocampal neurons	Prevention of Aβ25–35-induced cell death.	
		Swiss mice injected with Aβ25–35 Aged APP (SW)/tau (VLW) mice	Reversal of short-term visual episodic memory deficit.	

**Table 2:** Inhibition of DYRK1A as a therapeutic approach for diabetes and AD.

Molecular Therapeutic Target	Disease	Experimental Model	Main Findings	References
DYRK1A Inhibition	Diabetes	R7T1 mouse β cells and rat and human islets	Induction of β-cell proliferation.	[249]
		Diabetic mice transplanted with human islets	Stimulation of β-cell proliferation, increase in β-cell mass and insulin content. Enhancement in glucose tolerance	
		Rat and human β cells	Stimulation of β-cell proliferation. Upregulation of key β-cell transcription factors: PDX1, NKX6.1, and MAFA.	[252]
		Partial pancreatectomy mouse model	Regeneration of β-cell mass.	
		Diabetic NODSCID mice transplanted with marginal mass of human islets	Improvement in glycemic control.	
		Human β cells; NGS mice transplanted with human islets	Increased human β-cell proliferation. Enhancement in glucose-stimulated insulin secretion.	[250]
		INS-1 cells	Induction of cell proliferation. Preservation of insulin secretion functionality.	[263]
		*db/db* mice	Increase in β-cell proliferation. Reduction in fasting blood glucose levels and im- provement in glucose tolerance.	
	AD	HEK293 cells, SH-SY5Y neuroblastoma cells, and rat primary cortical neurons	Reduced tau phosphorylation at several AD-phos- phoepitopes.	[236]
		Neuronal cells	Reduction in tau hyperphosphorylation on serine 396 and AT8 epitope induced by soluble Aβ42 peptide.	
		HEK293 cells overexpressing APP	Normalization of Aβ production.	
		3xTg-AD mice	Reduction in APP and insoluble tau phosphorylation. Increase APP turnover in lysosomes, leading to reduced insoluble Aβ40 and Aβ42 levels. Improvement in reference and working memory.	[234]
		Aged APP/PS1 mice	Reduction in phospho-STAT3α levels and inflammatory cytokine release. Recruitment of microglial cells involved in Aβ clearance. Partial rescue of impaired synaptic plasticity and learning and memory deficits.	[264]
		AD–DS drosophila models overexpressing human tau, human Aβ, or minibrain	Reduced phosphorylation of human tau at Serine 262 Extension of drosophila shortened lifespan; im-- provement of locomotion performance and rescued memory and cognition deficits	[265]

## Data Availability

Not applicable.

## Abbreviations

[^18^F]-FDG	18-fluorodeoxyglucose
ABCB1	ATP-binding cassette subfamily B member 1
AD	Alzheimer’s disease
AMPA	α-amino-3-hydroxy-5-methyl-4-isoxazolepropionic acid
ApoE	apolipoprotein E
APP	amyloid precursor protein
ASF	alternative splicing factor
Aβ	amyloid beta peptide
BACE-1	β-site APP-cleaving enzyme 1
BBB	blood-brain barrier
BDNF	brain-derived neurotrophic factor
CNS	central nervous system
CSF	cerebrospinal fluid
DPP4	dipeptidyl peptidase 4
DS	Down syndrome
DYRK1A	dual-specificity tyrosine phosphorylation-regulated kinase 1A
GABA	gamma-aminobutyric acid
GAPDH	glyceraldehyde-3-phosphate dehydrogenase
GLP-1	glucagon-like peptide-1
GLUT	glucose transporter
GSK3β	glycogen synthase kinase 3β
HFD	high-fat diet
i.c.v	intracerebroventricular
IAPP	islet amyloid polypeptide
IDE	insulin-degrading enzyme
IDF	International Diabetes Federation
IGF	insulin-like growth factor
IRS	insulin receptor substrate
ISF	interstitial fluid
JNK	c-Jun N-terminal kinase
LRP1	low-density lipoprotein receptor-related protein 1
NFTs	neurofibrillary tangles
NMDA	N-methyl-D-aspartate
PET	positron emission tomography
PHF	paired helical filaments
PP2A	protein phosphatase 2A
PPAR	peroxisome proliferator-activated receptor
PSD95	postsynaptic density protein 95
PSEN1	presenilin 1
PSEN2	presenilin 2
ROS	reactive oxygen species
sAPPα	soluble APPα fragment
T2D	type 2 diabetes
Tau	tubulin-associated unit
Thr	threonin
TNF-α	tumor necrosis factor alpha
TrkB	tropomyosin receptor kinase B
WHO	World Health Organization
